# Emergency Department (ED), ED Observation, Day Hospital, and Hospital Admissions for Adults with Sickle Cell Disease

**DOI:** 10.5811/westjem.2017.9.35422

**Published:** 2018-02-12

**Authors:** David M. Cline, Susan Silva, Caroline E. Freiermuth, Victoria Thornton, Paula Tanabe

**Affiliations:** * Wake Forest School of Medicine, Winston-Salem, North Carolina; † Duke School of Nursing, Duke University Medical Center, Durham, North Carolina; ‡ Duke University Medical Center, Department of Surgery, Division of Emergency Medicine, Durham, North Carolina; § Duke University Medical Center, Department of Medicine, Division of Hematology, Durham, North Carolina

## Abstract

**Introduction:**

Use of alternative venues to manage uncomplicated vaso-occlusive crisis (VOC), such as a day hospital (DH) or ED observation unit, for patients with sickle cell anemia, may significantly reduce admission rates, which may subsequently reduce 30-day readmission rates.

**Methods:**

In the context of a two-institution quality improvement project to implement best practices for management of patients with sickle cell disease (SCD) VOC, we prospectively compared acute care encounters for utilization of 1) emergency department (ED); 2) ED observation unit; 3) DH, and 4) hospital admission, of two different patient cohorts with SCD presenting to our two study sites. Using a representative sample of patients from each institution, we also tabulated SCD patient visits or admissions to outside hospitals within 20 miles of the patients’ home institutions.

**Results:**

Over 30 months 427 patients (297 at Site 1 and 130 at Site 2) initiated 4,740 institutional visits, totaling 6,627 different acute care encounters, including combinations of encounters. The range of encounters varied from a low of 0 (203 of 500 patients [40.6%] at Site 1; 65 of 195 patients [33.3%] at Site 2), and a high of 152 (5/month) acute care encounters for one patient at Site 2. Patients at Site 2 were more likely to be admitted to the hospital during the study period (88.4% vs. 74.4%, p=0.0011) and have an ED visit (96.9% vs. 85.5%, p=0.0002). DH was used more frequently at Site 1 (1.207 encounters for 297 patients at Site 1, vs. 199 encounters for 130 patients at Site 2), and ED observation was used at Site 1 only. Thirty-five percent of patients visited hospitals outside their home academic center.

**Conclusion:**

In this 30-month assessment of two sickle cell cohorts, healthcare utilization varied dramatically between individual patients. One cohort had more hospital admissions and ED encounters, while the other cohort had more day hospital encounters and used a sickle cell disease observation VOC protocol. One-third of patients sampled visited hospitals for acute care outside of their care providers’ institutions.

## INTRODUCTION

Despite the existence of treatment guidelines for vaso-occlusive crisis (VOC) for patients with sickle cell anemia^1^ and evidenced-based summaries of treatment to guide emergency physicians,^2^ there is tremendous variability in the management of this disorder, the most common complication of sickle cell disease.^1^ The guidelines published by the National Health Lung, Blood Institute (NHLBI), and endorsed by the American Academy of Emergency Medicine, detail an acute pain algorithm.^1^ When possible, the NHLBI guidelines recommend treating pain associated with VOC using patient-specific protocols, as well as patient-controlled analgesia, aggressively treating pain plus reassessing the patient’s pain and level of sedation every 15–30 minutes. The NHLBI acute pain algorithm recommends treating acute pain in a day hospital (DH) or another short-term stay hospital setting such as an observation unit first, before considering hospital admission for uncomplicated VOC.^1^ The need for frequent hospital admission for patients with SCD and its association with premature death has been cited as a major concern for these patients.^3^,^4^,^5^

Readmission for patients with SCD within 30 days of the index visit has been cited as a concern.^5^ The U.S. 30-day readmission rate for Medicare patients with an index admission averaged 18.4% in 2012, down from 19% in the five years prior.^6^ The Centers for Medicare and Medicaid are beginning to monitor and will ultimately penalize hospitals for excess re-admissions within 30 days for the same diagnosis. In 2010, sickle cell anemia ranked number one, at 31.9%, for the percentage of patients readmitted within 30 days of an index visit.^7^

Opportunities to decrease admissions for patients with VOC have been demonstrated by use of a DH model.^8^–^13^ In this model, a hospital will dedicate space and staffing to provide care for patients experiencing a VOC, outside of the emergency department (ED) or an inpatient bed. While a feasible model, there are logistical issues to implementing this model on a national level, especially for small hospitals. Alternatively, 36% of hospitals in the U.S. have implemented an ED observation unit (EDOU),^14^ which may prove an alternative to hospital admission and treatment in a DH. The use of an EDOU for the treatment of VOC has been recommended for patients with continued pain, but without another indication for hospital admission.^1^,^15^,^16^ Transferring patients from an ED to the EDOU allows for more time to resolve the VOC and possibly avoid hospital admission.

In the context of a two-center quality improvement (QI) project designed to implement best practices for the ED management of patients with complications of sickle cell disease,^17^,^18^ we had a unique opportunity to prospectively examine the impact of different utilization rates of DH care, and/or EDOU care on rates of hospital admission for patients with sickle cell anemia experiencing VOC. We also had the opportunity to assess ED encounters and hospitalizations for our two cohorts of patients at outside centers within a 20-mile radius of each study site, an aspect of care rarely reported on.

The objectives of this prospective study were to 1) estimate and contrast the acute healthcare (ED visits, DH visits, ED observation and hospitalizations) utilization of two patient cohorts with sickle cell anemia presenting to one of two academic medical centers, and 2) assess acute care utilization of these cohorts seeking care at outside hospitals, within 20 miles of the home specialty centers.

**Population Health Research Capsule**

What do we already know about this issue?

Guidelines recommend day hospital or ED observation unit care rather than hospital admission to manage uncomplicated vaso-occlusive crisis pain for patients with sickle cell disease (SCD).

What was the research question?

What are acute healthcare utilization patterns of two different cohorts of patients with SCD?

What was the major finding of the study?

One cohort of SCD patients had more ED visits and hospital admissions; the other had more day hospital and ED observation unit visits.

How does this improve population health? *Institutions can consider ways to implement day hospital or ED observation unit care for patients with vaso-occlusive crisis pain to reduce hospital admissions.*

## METHODS

### Design and Procedures

Data for this project were reported in the context of a two-center QI project.^17^,^18^ Details of the project have been published.^17^–^20^ Briefly, the project attempted to optimize the treatment of VOC in the ED using best practices,^1^ while monitoring healthcare utilization and psychosocial referral needs and practices.^17^–^19^ In this paper, we report acute healthcare utilization data only (excluding the number of clinic visits).

### Institutional Review Board Approval

The study was approved by the institutional review boards at each of the two study sites. A waiver of consent was obtained at each site to examine ED, DH, ED observation encounters and hospital admissions for all patients with SCD during the study period. A subset of patients had the opportunity to sign consent for participation in an interview regarding care received (results not reported here); and relevant to this report, they had the option of providing consent for the study team to request healthcare use data (ED encounters and hospital admissions) from hospitals within 20 miles of each study site.

### Setting

We conducted our study at two urban EDs in the southeastern U.S., each affiliated with an academic medical center and with an emergency medicine residency training program. Characteristics of study site institutions and the sickle cell populations they serve are listed in Table 1. Differences between the study sites include the following: Site 1 used patient-controlled anesthesia in the ED, while Site 2 did not; Site 1 had a sickle-cell VOC observation unit protocol, while Site 2 did not.

### Sample

For 30 months at each institution, all ED records, DH and admission records for patients with SCD (any patient with *ICD-9* diagnosis codes 282.60–282.69, including any SCD complications, such as 517.3 acute chest syndrome, 289.52 splenic sequestration) were assessed for acute care visits. During the study, the Site 1 SCD clinic population averaged 500; at Site 2, the SCD clinic population averaged 195 (695 total for both sites). We included all ED visits and hospital admissions for patients, regardless of whether they were a SCD clinic patient or unknown to the clinic. Patients were recruited during an ED visit or hospitalization for enrollment in the above-mentioned larger QI project. The method used to recruit patients to sign informed consent to monitor outside hospital utilization identified patients based on the first three months of acute care utilization in an effort to balance the number of patients with high (≥ 5 visits), medium (3–4 visits), and low (≤2 visits) utilization patterns.

### Measures

Our study period was 30 months from October 2011 through March 2014. We assessed all acute care encounters (excluding clinic visits) for patients with sickle cell disease for acute complications of disease, including all ED encounters, EDOU encounters, DH encounters, and hospital admissions.

### Statistics

We analyzed the data using SAS 9.2 (Cary, NC). We conducted a Z test for equality of proportions to assess for differences in the sickle cell populations of the study sites.

### Acute Care Utilization, Home Institutions

We defined acute care utilization \as the total number of ED encounters, ED observation encounters, DH encounters, and hospital admissions for all patients with SCD. Study site research associates abstracted this data for the study period. If a patient had an ED encounter that resulted in a hospitalization, this accounted for two encounters. If a patient with an ED visit was transferred from the ED to observation under pain management protocol and then admitted, this accounted for three encounters. We purposefully counted each “setting” during the same date as a separate encounter to more comprehensively describe use.

### Acute Care Utilization, Outside Institutions

A total of 113 patients signed consent to have outside records assessed. We contacted all hospitals within 20 miles of each study site to obtain the number of acute care encounters (ED encounters or hospitalizations) for consented patients during the 30-month study period. No hospital within 20 miles of the home institutions had a DH or EDOU.

## RESULTS

### Acute Health Care Utilization for Regular Sickle Cell Clinic Patients

Over the 30-month study period, 427 patients (297 at Site 1 and 130 at Site 2) had acute care encounters. Demographics of the 427 patients seeking acute care are listed in Table 2; the median age was 30 years, but a small number of patients were of advanced age, up to 86 years. Hispanics represented 2.1% of patients. These 427 patients made 4,740 institutional visits to one of the two study sites, totaling 6,627 different acute care encounters (ED encounters, DH encounters, EDOU encounters, hospital admissions, or combinations of these encounters. The range in total number of acute care encounters per site over the 30-month period, including any of the four different encounter types, ranged from a low of 0 (203 of 500 patients [40.6%] at Site 1; 65 of 195 patients [33.3%] at Site 2), and a high of 152 (5/month) acute care encounters for one patient at Site 2.

### Acute Health Care Utilization for Patients with Acute Care Encounters

The number of unique patients that had acute care encounters at Site 1 was more than twice the number at Site 2 (297 vs 130), yet the number of hospital admissions was close between sites (983 at Site 1, and 887 at Site 2). The number of ED encounters was greater at Site 2 than Site 1 (1,688 at Site 2 vs. 1,596 at Site 1) despite Site 2 having 45% the number of unique patients. Site 1 used the DH more than Site 2, 1,207 encounters at Site 1 (8.5 visits/patient for 142 patients using the DH at Site 1) vs. 199 encounters at Site 2 (4.4 visits/patient for the 50 patients using the DH at Site 2). Totaling the number of acute care encounters (summing individual components of the different service areas during the study period), Site 1 had fewer acute care encounters per patient, 13.0 versus 21.3 acute care encounters per patient at Site 2, over 30 months.

Table 3 compares the access patterns between the sickle cell populations at each institution. For the group that had at least one acute care encounter during the study period, patients at Site 2 were more likely to be admitted during the study period (88.4% vs. 74.4%, p = 0.0011) and have an ED visit (96.9% vs. 85.5%, P = 0.0002). The percent of patients having at least one DH encounter between sites was not significantly different (47% at Site 1 vs. 38.4% at Site 2, p = 0.073). However, when comparing the number of DH visits of each site per patient requiring acute care encounters, DH was used more atsite 1 (1,207 visits /297 patients= 4.1 visits per patient) than for Site 2 (199 visits /130 patients = 1.5 visits per patient).

### Hospital Admission Following a Primary Encounter

Table 4 contrasts the rates of admission following a primary encounter type (excluding the 20 direct admissions [no ED Visit] for Site 1, and 49 direct admissions [no ED Visit] for Site 2 that occurred during the study period). The hospital admission rate was less common following all primary encounters at Site 1 (34%) vs. Site 2 (44%). However, the admission rate following an ED visit was slightly less at Site 2 (47%) vs. Site 1 (53%). Site 2 did not have an ED observation protocol at the time of the study; the admission rate following placement in the EDOU at Site 1 was lower than the admission rate following an ED encounter at either site, at 36%. The hospital admission rate was low following a DH encounter at Site 1: 8%, compared to 24% at Site 2.

### Outside Hospital Utilization

We obtained consent from 113 patients to have outside hospitals within 20 miles of their home academic centers queried for medical records of acute care encounters, 56 at Site 1, and 57 at Site 2. For this consented group, there were 190 outside-hospital ED encounters involving 38 patients (5/patient), and 110 outside hospitalizations involving 27 patients (4/patient). These 300 acute care encounters represented 40 of the 113 consented patients; therefore, 35.4% of the patients consented visited hospitals outside their home academic centers for some of their acute care needs. Table 5 compares ED visits and hospital admissions per patient per year; patients from Site 1 had more outside hospital encounters than patients from Site 2.

## DISCUSSION

To the best of our knowledge, this is the first report of healthcare utilization in patients with SCD that includes DH and ED observation visits, in addition to the routinely reported ED visits and hospitalizations. We intentionally “counted” each encounter, and the numbers are significant. We attempted to dissect the “locations” in an effort to more fully understand all healthcare use for treatment of VOC and to begin to understand the potential for all locations as alternatives for treatment of VOC.

During the project, several changes at both sites affected healthcare utilization options. Immediately prior to the onset of the study, Site 1 opened a new day hospital, enabling the management of mild episodes of VOC crisis with a DH stay; in contrast, at Site 2, the main provider that admitted patients to the DH took an 18-month medical leave, temporarily limiting the use of the DH at Site 2.

Therefore, it is not surprising that the DH was used more for Site 1 patients needing acute pain management of VOC (1,207 encounters for 297 patients at Site 1, vs. 199 encounters for 130 patients at Site 2). It should be noted that the percentage of patients with one or more encounters to the DH at each site was not significantly different. The difference in usage reflected the frequency of DH use per patient during the study period (an average of 4 encounters/patient at Site 1, vs.1.5 encounters/patient at Site 2) rather than the percentage of patients with at least one DH encounter at each site. Another difference in management style is reflected in the hospital admission rate following a DH encounter between sites. Site 1 had a low post-DH encounter hospital admission rate, 8%, compared to Site 2, 24%.

Dedicated DH management of patients with SCD has been shown to reduce full hospital admissions and total costs.^11^,^12^, ^21^ It is clear there was a lower threshold for admission from the DH at Site 2 when compared with Site 1. This reflects practice pattern differences. Emergency physicians should work with area hematologists to explore expanded use of DH treatment of uncomplicated VOC to reduce hospital admission for those cases where hospital admission is not otherwise warranted.

We also report the use of an EDOU to help decrease hospital admission. Site 1 placed 67 patients in the observation unit rather than admitting to the hospital after inability to discharge after the ED stay; the admission rate was less than the SCD-VOC ED admission rate (36% verses 53%). A Brazilian hospital center successfully implemented an EDOU protocol and reduced hospital admissions; however, generalization of findings is limited to the small sample size, as there were less than 30 hospital admissions for sickle cell crisis each year.^22^ Two studies proclaiming a 50–55% reduction in hospital admission rates following implementation of a dedicated SCD-VOC observation protocol have been published in abstract form,^15^,^16^ but the detailed reports have yet to be published. Additional details are required before conclusions can be generalized to other settings. However, emergency physicians with access to an EDOU should consider establishing a SCD-observation protocol to reduce hospital admissions for uncomplicated VOC.

Few prior studies assessed sickle cell patients’ use of hospital facilities outside of their specialists’ home institutions. Our finding of 34.5% of SCD patients visiting outside institutions is slightly less than that found by Woods et al. in 1997, who found 39% of SCD patients in the Illinois statewide database used more than one hospital for care.^23^ However, our finding of 34.5% outside hospital use is considerably less than Panepinto et. al. study using a database from eight states, which found that 48.7% of adult patients with SCD used more than one hospital.^24^ The fact that our patients had access to a hematologist for regular care may have reduced their need to seek care outside of the home institutions, while the other two cited studies reflected a more general SCD patient population, likely with less hematology follow-up care. Furthermore, patients seeking care elsewhere may represent needs unmet by the home institution.^25^ Our findings highlight the importance of measuring the cost of outside hospital utilization when studying the financial impact of new treatments or programs initiated at the investigator’s institution.

While the majority of patients with sickle cell disease at each study site presented for acute care during the study period, a significant number had no acute care encounters, for a period longer than previously reported previously.^26^, ^27^ Approximately 40% of clinic patients at Site 1, and 33% of clinic patients at Site 2 had no acute care encounters at their hematologist’s institution, or at hospitals within 20 miles of the hematologist, during the 2.5-year monitoring period. Our findings should be compared to an eight-state study of statewide inpatient and ED databases that found only 29% of patients had no acute care encounters related to their sickle cell anemia over a 12-month period.^26^ Darbari et. al. reported percentages similar to our study, 40%, but the assessment period was only one year.^27^ Our findings document 33–40% of two populations of patients with sickle cell disease being managed by hematologists without the need for acute care encounters for period of 30 months. We believe this is an important finding and further refutes the commonly held myth that all patients with SCD are high utilizers.

Another important finding is that a greater proportion of patients at Site 2 had one or more hospital admissions (88.4% at Site 2 vs. 74.4% at Site1), and had one or more ED visits (96.6% at Site 2 vs. 85.5% at Site 1). Furthermore, while the number of patients with acute care encounters at Site 1 was more than twice the number at Site 2 (297 vs. 130), Site 2 patients had more total ED encounters than Site 1 patients (1,688 vs. 1,596 encounters). This again speaks to differences in practice patterns between sites that can be guided with strong input from the patients’ hematologists.

It has been documented previously how a minority of patients with SCD account for a disproportionately greater number of encounters;^26^–^31^ however, the variation in acute care usage between sickle cell populations has not been demonstrated previously within a single study. Clearly, the patients at Site 2 had more acute care encounters per patient (21.3 per patient at Site 2, vs. 13.0 per patient at Site 1). Our study did not assess the differences in methods of sickle cell disease management in the outpatient clinics; future study should investigate differences in all management methods, as well as differences in the patient characteristics, to determine the cause of this difference in acute care utilization.

## LIMITATIONS

Our study was a prospective observational study, and we did not randomize patients to any specific treatment plan or setting. Although it was our intention to provide optimal and uniform care at both sites, providers at Site 2 were unable to initiate patient-controlled analgesia in the ED. However, use of patient-controlled analgesia at Site 1 had unique problems, including delays to initiation of pain treatment as the device takes more time to set up than simple, single intravenous injection of pain medicine. Patient satisfaction with pain medication (reported previously) was not significantly different between sites.^17^

We did not assess outside hospital use beyond a 20-mile radius of each study site. We learned from discussions with patients that a few had received acute medical care at facilities outside of the 20-mile radius surrounding the home institutions, but we are not able to quantify or comment further on this care as patients were consented for hospitals only within the 20-mile radius. We observed differences in management styles, but we were unable to determine from this data to what extent the differences we observed were due to physician practice, patient disease severity, or other factors. Each site experienced a deficit in hematologist specialty coverage that reduced the use of the DH until a replacement could be found (three months at Site 1, 18 months at Site 2). Our patient population had access to a hematology specialty clinic during the entire study period; our finding may not be applicable to settings without readily available hematology follow-up^32^ and hematologist-directed day hospital management for patients with sickle cell disease.

## CONCLUSION

In this 30-month assessment of two sickle cell clinic cohorts, healthcare utilization varied dramatically between individual patients, with no acute care encounters for 33–40% for the two clinic cohorts and a high of five encounters per month for 30 months for one patient at Site 2. One cohort had more hospital admissions and ED encounters, while the other cohort had more day-hospital encounters. The admission rate following an acute care encounter was lower for the site that had fewer ED encounters and hospital admissions per patient. One third of patients visited hospitals for acute care outside of their care providers’ institutions.

## Figures and Tables

**Table 1:** Characteristics of study-site institutions and the sickle cell populations they serve.

Characteristic	Site 1	Site 2
Sickle cell disease (SCD) Specialist care	Comprehensive SCD Center with hematologist outpatient services and inpatient care	Comprehensive SCD Center with hematologist outpatient services and inpatient care
Adult ED volume	66,000 visits, year 2014	78,000 visits, year 2014
Inpatient beds, year 2014	919 beds	885 beds
Day hospital hours of operation for sickle cell patients with VOC	8 Hours, 9 to 5 PM, weekdays only.	8 hours, 9 AM to 5 PM, weekdays only
ED observation unit care for patients with SCD VOC	ED Observation unit in place, dedicated observation protocol for treatment of uncomplicated VOC	ED Observation unit in place; however, no dedicated VOC observation protocol in place
Patient specific treatment plans for pain control	In place at time of study initiation	Established during first 6 months 30-month study period
Patient controlled analgesia (PCA)	PCA protocol available in the ED	No PCA use in the ED
Regular sickle cell clinic patients	500 patients	195 patients
SCD Clinic patients with no hospitalizations, ED Visits, or day hospital visits over 2.5 years	203 patients, 40.6%	65 patients, 33.3%

*ED,* emergency department; *VOC*, vaso-occlusive crisis.

**Table 2:** Patient demographics

Parameter	Site 1 N=297	Site 2 N=130	Overall N=427
Age in Years	Mean; Median (range)	Mean; Median (range)	Mean; Median (range)
			
	32.7 years; 30 (18–78)	32.1 years; 29 (18–86)	32.5 years; 30 (18–86)
Race	N (%)	N (%)	N (%)
			
Black	286 (96.3%)	129 (99.2%)	415 (97.2%)
White	3 (1%)	0	3 (0.7%)
American Indian	1 (0.3%)	0	1 (0.2%)
Unknown	7 (2.4%)	1 (0.8%)	8 (1.9%)

**Table 3:** Access patterns of sickle-cell patients with at least one encounter type.

Encounter type	Unique patients with at least one of each encounter type	Site 1, N= 297	Site 2, N=130	P value
Hospital admissions	336	221 (74.4%)	115 (88.4%)	0.0011*
ED visits	377	251 (85.5%)	126 (96.9%)	0.0002*
Day hospital visits	192	142 (47.8%)	50 (38.4%)	0.0735
ED observation unit stays	48	48 (16.1%)	0	

*ED,* emergency department.

**Table 4:** Admission rates following ED, day hospital, or ED observation encounters at each site.

Encounter types	Site 1 encounters followed by hospital admission	Site 2 encounters followed by hospital admission	P value±
Emergency department	824/1529 (54%)	791/1688 (47%)	0.0222
Day hospital	91/1207 (8%)	47/199 (24%)	0.0001
ED observation unit	24/67 (36%)	0	
Totals*	939/2803 (33%)	838/1887 (44%)	0.0001

± Chi square.

* These totals do not reflect the 20 direct admissions for Site 1, and 49 direct admissions for Site 2.

**Table 5:** Outside hospital admissions and ED visits for a subset of consented patients.

	Site 1	Site 2
Subset of patients consented for survey of outside hospital* utilization, 113 total patients consented and surveyed	56 patients consented	57 patients consented
190 outside ED visits involving 38 of 113 patients, 33.6% had outside ED visits.	Average of 2.8 ED visits per patient per year over 2.5 years	Average of 0.6 ED visits per patient per year over 2.5 years
110 outside hospitalizations involving 27 of 113 patients, 23.9% had outside hospital admissions)	Average of 1.6 hospitalizations per patient per year over 2.5 years	Average of 0.4 hospitalizations per patient per year over 2.5 years

*ED*, emergency department.

* Acute health care encounters for enrolled patients at hospitals within 20 miles of each of the two study sites
